# Insight from sirtuins interactome: topological prominence and multifaceted roles of SIRT1 in modulating immunity, aging, and cancer

**DOI:** 10.5808/gi.23003

**Published:** 2023-06-30

**Authors:** Nur Diyana Zulkifli, Nurulisa Zulkifle

**Affiliations:** Department of Biomedical Sciences, Advanced Medical and Dental Institute, Universiti Sains Malaysia, Bertam, 13200 Kepala Batas, Penang, Malaysia

**Keywords:** aging, cancer, immunity, protein interaction network, sirtuins

## Abstract

The mammalian sirtuin family, consisting of SIRT1-SIRT7, plays a vital role in various biological processes, including cancer, diabetes, neurodegeneration, cardiovascular disease, cellular metabolism, and cellular homeostasis maintenance. Due to their involvement in these biological processes, modulating sirtuin activity seems promising to impact immune- and aging-related diseases, as well as cancer pathways. However, more understanding is required regarding the safety and efficacy of sirtuin-targeted therapies due to the complex regulatory mechanisms that govern their activity, particularly in the context of multiple targets. In this study, the interaction landscape of the sirtuin family was analyzed using a systems biology approach. A sirtuin protein-protein interaction network was built using the Cytoscape platform and analyzed using the NetworkAnalyzer and stringApp plugins. The result revealed the sirtuin family's association with numerous proteins that play diverse roles, suggesting a complex interplay between sirtuins and other proteins. Based on network topological and functional analysis, SIRT1 was identified as the most prominent among sirtuin family members, demonstrating that 25 of its protein partners are involved in cancer, 22 in innate immune response, and 29 in aging, with some being linked to a combination of two or more pathways. This study lays the foundation for the development of novel therapies that can target sirtuins with precision and efficacy. By illustrating the various interactions among the proteins in the sirtuin family, we have revealed the multifaceted roles of SIRT1 and provided a framework for their possible roles to be precisely understood, manipulated, and translated into therapeutics in the future.

## Introduction

Sirtuins are a highly conserved family of proteins that play critical roles in various biological processes, including immunity, aging, and cancer. In the 1990s, the discovery of the silent information regulator 2 (Sir2) gene in yeast, which was found to be required for lifespan extension in response to calorie restriction, sparked interest in sirtuins and their potential impact on human health [1]. Sirtuins are NAD+-dependent deacetylases that modulate chromatin structure and protein function through their deacetylase activity, leading to changes in gene expression and cellular behavior [2]. In humans, seven sirtuin members were identified: SIRT1, SIRT2, SIRT3, SIRT4, SIRT5, SIRT6, and SIRT7 [3], each with unique functions and subcellular localizations [4]. For example, SIRT1 is predominantly located in the nucleus and regulates gene expression through deacetylation of histones and transcription factors, whereas SIRT3 is primarily found in the mitochondria and controls metabolic processes through deacetylation of metabolic enzymes [5,6].

SIRT1 has been shown to play a critical role in the differentiation and function of regulatory T cells, which are key immune cells that help maintain immune tolerance and prevent autoimmune diseases [7]. Moreover, it has been demonstrated to play a vital role in extending lifespan in various model organisms, including yeast, flies, and mice [8-11], and has been implicated in regulating age-related diseases, such as neurodegenerative disorders, metabolic disorders, and cardiovascular disease [12]. In the context of cancer, the role of SIRT1 is significant, but its specific function is still a subject of debate as its effect can vary depending on the cellular environment, target proteins in specific signaling pathways, or cancer types, and can either act as a tumor suppressor or promoter [13]. While SIRT1 has been the most extensively studied member of the family, other members such as SIRT3, SIRT6, and SIRT7 have also been found to play important roles in immunity, aging, and cancer [14-16].

Due to their involvement in these biological processes, modulating sirtuin activity has the potential to impact the pathways related to immune- and aging-related diseases, as well as cancer [17]. However, despite the promise shown in preclinical studies, much remains unknown about the safety and efficacy of sirtuin-targeted therapies [18]. Although progress has been made in understanding the roles of sirtuins in disease pathogenesis, there is still much to learn about the complex regulatory mechanisms that govern their activity, particularly in the context of multiple targets and systems. Therefore, in this study, we take a systems biology approach to examine the intricate and multifaceted roles of sirtuins in modulating immunity, aging, and cancer. Specifically, we use a protein-protein interaction (PPI) network analysis to gain insights into the sirtuins’ interaction landscape and the topological prominence of SIRT1, the most extensively studied sirtuins, and its interactions with other key proteins in these biological processes. We aim to provide a comprehensive understanding of the mechanisms by which SIRT1 influences immune response, aging, and cancer development. The systems-level analysis also seeks to shed light on the functional diversity of sirtuins and their potential as therapeutic targets for immune- and aging-related diseases as well as cancer. By revealing the intricate and complex interplay between sirtuins and other proteins in these processes, our study provides a foundation for the development of novel therapies that can target sirtuins with precision and efficacy.

## Methods

### Data retrieval and network construction

Sirtuins PPI network was constructed using Cytoscape ver3.6.0 [19] and the UniProt accession numbers of all sirtuins as queries. The sirtuins direct interaction partners were extracted from experimentally derived protein interaction databases IntAct, MINT, and IMEx consortium [20-22] using the Cytoscape plugin PSICQUIC web service client [23]. To obtain a reliable network, all nodes in the integrated PPI network were filtered to only include *Homo sapiens* species, and redundant nodes and edges, as well as self-interactions were removed. Interconnectivity among proteins in the sirtuins PPI network was identified by submitting the list of proteins as queries on stringApp plugin [24] with the confidence score cutoff set at 0.7 and the maximum number of additional interactors set at zero.

### Annotating aging-, immune-, and cancer-associated genes on sirtuins PPI network

In order to identify proteins associated with immune response, aging and cancer from the sirtuin PPI network, a list of genes related to each condition along with their detailed annotations was obtained from InnateDB, HAGR’s GeneAge and COSMIC Cancer Gene Census database ver90 [25-27]. The list was then imported into the node data column of sirtuin PPI network from a tabular file.

### Network analysis

The network's hub and bottleneck nodes were identified by analyzing the degree and betweenness centrality (BC) value of each node using NetworkAnalyzer [28]. Network clustering and functional enrichment analysis were performed using the stringApp plugin. For clustering, stringApp used the clusterMaker2 algorithm to run Markov clustering (MCL) [29-30] with a granularity parameter value set at 4 and functional enrichment analysis was carried out with a false discovery rate (FDR) threshold of 5%.

## Results and Discussion

### Each sirtuin targets a specific set of protein

**Fig. 1.**
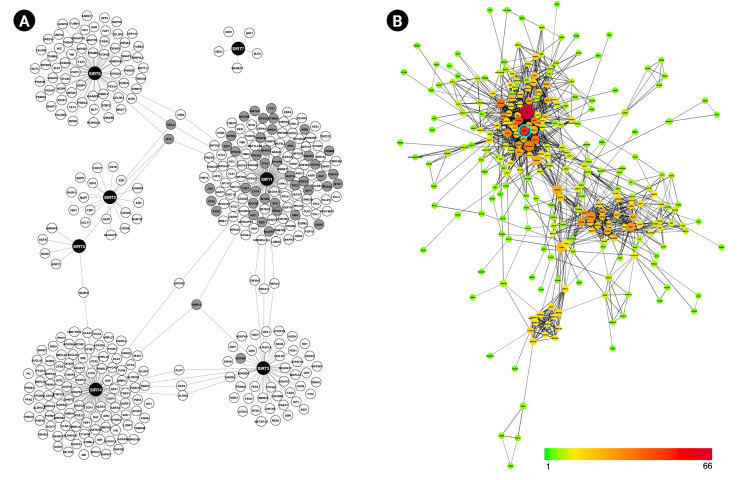
(A) Sirtuins protein-protein interaction network shows each sirtuin has an almost unique set of interaction partners. Grey nodes indicate that the genes are associated with immunity, aging and/or cancer. (B) Interconnectivity among proteins in (A): Nodes are colored according to the number of interactors, ranging from 1 (green) to 66 (red). The node size represents the value of BP; the bigger the node, the higher the value. BP, biological process.

Sirtuin members SIRT1, SIRT2, SIRT3, SIRT4, SIRT5, SIRT6, and SIRT7 have been implicated in various physiological and pathological processes such as genomic stability, gene expression, reprogramming, obesity, neurodegeneration, diabetes, and cancer [31]. To gain a better understanding of the interaction landscape of sirtuins and their targets, we set out to construct a PPI network of the sirtuin family using three resources: IntAct, MINT, and IMEx. Following network construction, data from InnateDB, GeneAge, and COSMIC were integrated into the network, resulting in a completely informed PPI network with 385 nodes and 395 edges (Fig. 1A). SIRT1 has the most interaction partners with 135 edges, followed by SIRT4, SIRT6, SIRT3, SIRT2, and the fewest, SIRT5 and SIRT7 with six and five edges, respectively. The network clearly demonstrates that each sirtuin is linked to a distinct set of proteins and rarely shares interaction partners. With the exception of SIRT7, the remaining sirtuins are nevertheless still linked indirectly through a variety of intermediate proteins.

The target specificity portrayed by each sirtuin is justified as each sirtuin serves a distinct function and takes part in a different biological and pathological event. For instance, SIRT1 is involved in metabolism and inflammation, whereas SIRT2 is involved in cell cycle and tumorigenesis. Like SIRT1, SIRT3 is also involved in metabolism while SIRT4 is involved in insulin secretion, SIRT5 in ammonia detoxification, SIRT6 in DNA repair, metabolism, and TNF secretion and lastly SIRT7 in rRNA transcription [32-35].

Sirtuins' diverse localization at different subcellular sites is a result of evolutionary divergence dating back to Archaea and contributes to the differences in their roles. These days, SIRT1, SIRT6, and SIRT7 are mostly found in the nucleus, while SIRT3, SIRT4, and SIRT5 are mostly found in the mitochondria, whereas SIRT2 is found mainly in the cytoplasm [36]. The target specificity and distinct localization of sirtuins are explained by variations in the sequence and length of their N- and C-terminal domains, which cause variations in their 3D structure, affecting their binding interfaces and, as a result, influencing their binding partners [32].

### Topological significance of SIRT1 in sirtuins PPI network

Next, the STRING database was used to analyze the interconnectivity of sirtuins PPI network, yielding 338 nodes and 1,038 edges. Topological network analysis suggested that SIRT1 is significantly important based on the node’s degree and BC measurement. As illustrated in Fig. 1B, the color scale from green to yellow to orange to red denotes the number of connections for each node (range, 1 to 66); thus, the more reddish nodes indicate the highest connectivity and are considered hub nodes. TP53 is the most connected node in the network, followed by SIRT1. Meanwhile, identification of bottleneck nodes using measurement of BC is indicated by node size increment; the larger the node, the higher the BC value. SIRT1 size is observed to be on the large side, trailing behind TP53, TUFM, HSPA9, HSP90AA1, ATP5A1, and HSPA8.

Highly networked, as evidenced by a node with a large number of interactors, implying that it regulates a wide range of cellular functions via its target. Meanwhile, a high BC value indicates that this protein serves as a bridge between network clusters more frequently than others. If each network cluster represents a distinct functional unit, SIRT1 is very likely to be involved in the crosstalk of several distinct pathways. This observation is critical when considering SIRT1 as a therapeutic target because such topological position in a network suggests that perturbing SIRT1 for one pathway may have unintended consequences for other pathways. Interestingly, while SIRT1 exhibited very prominent topological characteristics, other sirtuins, in contrast, did not.

The topological importance of SIRT1 in our PPI network should be taken with a grain of salt, as it may be the result of studies that focused on SIRT1 in the past. The PPI network was built using reported interactions from the database, which raises the possibility that other sirtuins' interactions are still not well-characterized. However, there is a strong possibility that our PPI network is reliable because studies have shown that SIRT1 has more disordered segments in its N- and C-terminal regions, implying that it is capable of forming broad and specific interactions with multiple proteins [33,37]. To obtain a fair analysis from a system biology standpoint, an equivalent amount of research on the remaining sirtuins must be conducted.

### Gene ontology terms and Kyoto Encyclopedia of Genes and Genomes pathways analysis

**Fig. 2.**
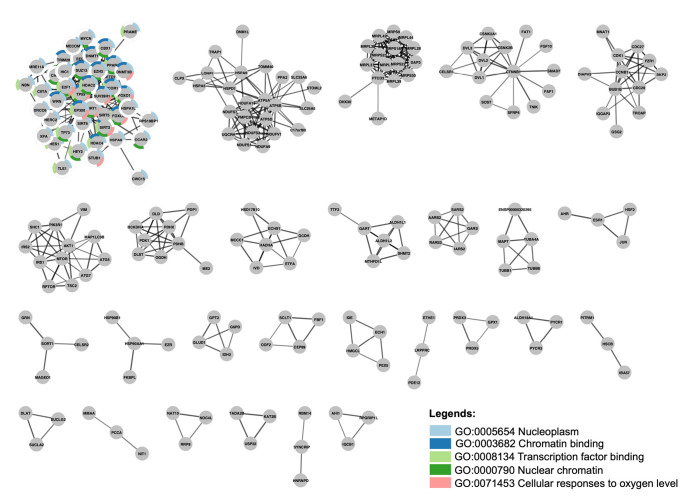
StringApp cluster analysis with clusters fewer than two nodes are omitted. Cluster 1 functional enrichment analysis revealed the top five gene ontology that are overrepresented in Cluster 1.

The stringApp cluster analysis extracted at least 27 clusters with at least three nodes. Cluster 1 is made up of SIRT1, SIRT2, SIRT5, SIRT6, and 40 other proteins, whereas SIRT3, SIRT4, and SIRT7 are not part of any cluster. Functional enrichment analysis was then performed to characterize Cluster 1, which consisted of SIRT1, SIRT2 SIRT5, and SIRT6 among the gene set, yielding a list of 159 statistically significant terms spanning four categories: gene ontology (GO) biological process (BP), GO molecular function (MF), GO cellular component (CC) and Kyoto Encyclopedia of Genes and Genomes pathways. Of these, the five most significant terms were nucleoplasm (CC) with FDR value 2.78 × 10^-24^, chromatin binding (MF) (FDR = 6.8 × 10^-15^), transcription factor binding (MF) (FDR = 6.4 × 10^-13^), nuclear chromatin (CC) (FDR = 2.16 × 10^-10^) and cellular response to oxygen (BP) (FDR = 3.35 × 10^-10^), which covered 42, 20, 18, 19 and 11 out of the 44 proteins in the cluster, respectively. Split donut charts were drawn around the nodes to show which proteins are annotated with which of these terms (Fig. 2).

SIRT1 and SIRT6 are primarily localized in the nucleus, where they play a role in regulating gene expression through their deacetylase activity on histones and transcription factors. This includes the regulation of chromatin structure and gene transcription in response to various stimuli such as oxidative stress, DNA damage, and nutrient availability, which are all related to the cellular response to oxygen. SIRT2, although mainly localized in the cytoplasm, can transiently enter the nucleus during mitosis [38]. Hypoxia is a well-established cellular response to low oxygen levels, which can trigger a range of physiological responses, including alterations in gene expression and chromatin structure. SIRT1 is a well-known regulator of HIF1α and HIF2α that either enhances or reduces HIF-dependent genes depending on the type of cell thereby influencing the cell’s hypoxic response [39]. SIRT2 also participates in the hypoxia-induced stress response by repressing the protective response and enhancing the toxicity caused by hypoxia [40].

### SIRT1 association with aging-, cancer-, and immune-associated genes

**Fig. 3.**
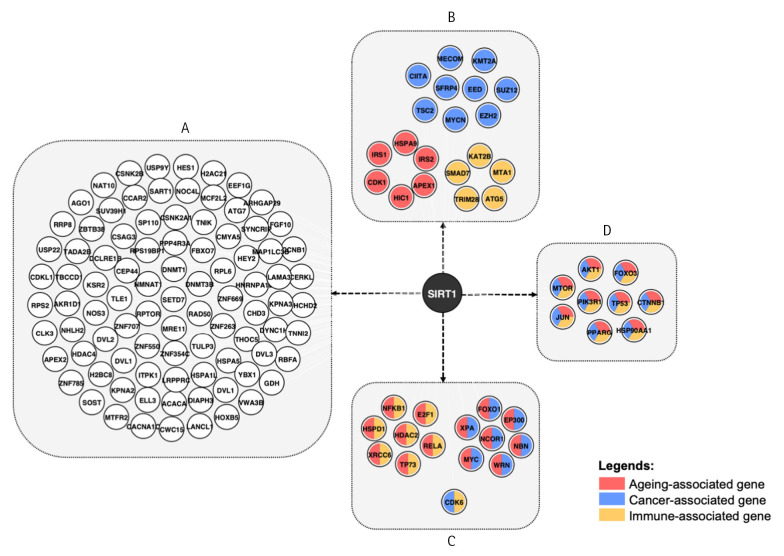
SIRT1 protein interaction partner: A, Proteins that are associated with neither aging, cancer nor immunity pathway; B, Gene involved in either aging, cancer, or immunity pathway; C, Genes involved in combination of the twopathways; D, Genes involved in all three pathways.

Going back to Fig. 1A, the integration of the network with aging, cancer, and immunity data revealed that 45 out of 385 nodes are aging-, cancer- and immune-associated genes, with 44 of them being SIRT1 interaction partners. In Fig. 3, the network demonstrates that while some modes are involved in one of the pathways, others act as multifaceted modulators, as indicated by color combination in each node. There are 29, 22, and 26 genes that are either exclusively or promiscuously associated with aging, immune, and cancer, respectively. Among these, nine are involved solely in cancer, five are immune-related and six are linked to aging. Whereby, 15 genes are observed to modulate two pathways, and nine genes are associated with all three pathways.

The network demonstrated an association of SIRT1 with cancer, innate immune response, and aging through eight different nodes, namely TP53, PIK3R1, PPARγ, FOXO3, mTOR, AKT1, CTNNB1, and HS9OAA1. Cancer, immune response, and aging are biological processes that are inextricably linked. Firstly, the immune system plays a crucial role in recognizing and eliminating cancer cells. Immune cells such as T cells, B cells, and natural killer cells are responsible for identifying and attacking cancer cells. However, aging causes the immune system's function to decline, making it less effective at this particular task, which is one of the reasons why cancer incidence increases with age [41]. Secondly, chronic inflammation, which is a hallmark of aging, can contribute to the development of cancer. Inflammatory cells produce reactive oxygen species and other damaging molecules that can damage DNA and other cellular components, leading to mutations and ultimately cancer. Thirdly, as we observed in our study, many of the same genes and pathways that regulate aging and immune response also play a role in cancer development.

The best-known cancer-related protein is p53, and its gene is thought to be the most frequently mutated gene in cancer. Several downstream targets, including p21, MDM2, GADD45, cyclin G, and Bax, which induce cell cycle arrest, are transcriptionally activated by p53, making it the cellular gatekeeper for cell growth and division [42]. The p53 status in cancer cells significantly impacts the immune response. Cells with p53 loss or mutation can affect the activity and recruitment of myeloid and T cells, allowing immune evasion and accelerating cancer growth [43]. There is also strong evidence of p53 associations in aging. According to Feng et al. (2007) [44], p53 response to γ-irradiation declines significantly in various tissues of aging mice due to a decrease in its protein stabilization after the stress. Furthermore, p53 also regulates AMPKβ1, TSC2, and PTEN that interact with the IGF-1–AKT–mTOR signaling pathway, which is involved in regulating cell growth and proliferation. p53 is reported to play a tissue-specific role in regulating these genes, and the loss of p53 function with age can contribute to the development of age-related diseases such as cancer [45].

PI3K/AKT pathway is one of the kinases that can activate p53, and PIK3R1 is a regulatory subunit of this pathway. Two important cellular processes regulated by PI3K/AKT are proliferation and apoptosis [46]. In this pathway, the phosphorylated PTK2 provides a binding site for the SH2 domain of the regulatory subunit (PIK3R1) of PIK3CA. Subsequent production of PI3,4,5-P3 provides a binding site for the PH domain of both PDK1 and AKT. After its activation by PDK1, AKT phosphorylates a large number of proteins that directly or indirectly regulate cell death, such as p53 and FOXO3. mTOR is also regulated by the PI3K/AKT pathway and is a downstream effector of AKT1. Dysregulation of the PI3K/AKT pathway can lead to immune dysfunction, such as autoimmunity and immunodeficiency, and contribute to the development of various diseases, including cancer [47].

PPARγ and SIRT1 can interact and modulate each other’s activity. PPARγ induces the expression of SIRT1, which in turn can deacetylate and activate PPARγ. Additionally, SIRT1 has been shown to inhibit the activity of PPARγ coactivator-1 alpha, which is involved in the regulation of mitochondrial biogenesis and energy metabolism [48]. The interaction between PPARγ and SIRT1 has been implicated in various physiological and pathological processes, including adipocyte differentiation, insulin sensitivity, lipid metabolism, inflammation, and cancer [49]. When cancer develops, cells' metabolisms must be reprogrammed to cope with a depleted supply of oxygen and nutrients to support their rapid proliferation and biomass production. A progressive rise of oxidative stress and related inflammatory reaction appears to be the hallmarks of the aging process and many age-related diseases [50].

Meanwhile, mutations in the *CTNNB1* gene, which encodes β-catenin, a crucial component of the Wnt signaling pathway, have been associated with early events in carcinogenesis as well as a scarcity of immune cells in the tumor microenvironment and a poor clinical response to immunotherapy [51,52]. Additionally, restoring Wnt/β-catenin signaling has shown promise as a therapeutic strategy for age-related diseases such as Alzheimer's disease by promoting enhanced synaptic plasticity, neuronal survival, and neurogenesis [53].

In a nutshell, the finding that p53, PIK3R1, PPARγ, FOXO3, mTOR, AKT1, and β-catenin interact with SIRT1 and are associated with cancer, aging, and immune response suggests that targeting one of these proteins could have effects on the others. Targeted therapy directed at a specific protein can have both beneficial and harmful effects, as it can disrupt the normal functions of these genes and their interactions in different pathways. For example, targeting p53 for cancer therapy may also inhibit the immune response, leading to increased risk of infections or other immune-related disorders. Therefore, the design of targeted therapy should consider the complex interplay between these genes and their roles in multiple cellular pathways. Further research is needed to fully understand the potential risks and benefits of targeted therapy for these proteins in cancer, aging, and immune-related diseases.
